# Oxidative Stress and Antioxidants in Uterine Fibroids: Pathophysiology and Clinical Implications

**DOI:** 10.3390/antiox12040807

**Published:** 2023-03-26

**Authors:** Abdelrahman AlAshqar, Bethlehem Lulseged, Akailah Mason-Otey, Jinxiao Liang, Umme Aoufa Mafruha Begum, Sadia Afrin, Mostafa A. Borahay

**Affiliations:** 1 Department of Obstetrics, Gynecology and Reproductive Sciences, Yale School of Medicine, New Haven, CT 06510, USA; 2 Department of Gynecology and Obstetrics, Johns Hopkins University School of Medicine, Baltimore, MD 21287, USA; 3 Department of Gynecology and Obstetrics, Khulna City Medical College Hospital, Khulna 9100, Bangladesh

**Keywords:** oxidative stress, uterine fibroids, antioxidants, hypoxia

## Abstract

In the last few decades, our understanding of the complex pathobiology of uterine fibroid development has grown. While previously believed to be a purely neoplastic entity, we now understand that uterine fibroids possess different and equally important aspects of their genesis. An increasing body of evidence suggests that oxidative stress, the imbalance between pro- and antioxidants, is an important factor in fibroid development. Oxidative stress is controlled by multiple, interconnecting cascades, including angiogenesis, hypoxia, and dietary factors. Oxidative stress in turn influences fibroid development through genetic, epigenetic, and profibrotic mechanisms. This unique aspect of fibroid pathobiology has introduced several clinical implications, both diagnostic and therapeutic, that can aid us in managing these debilitating tumors by using biomarkers as well as dietary and pharmaceutical antioxidants for diagnosis and treatment. This review strives to summarize and add to the current evidence revealing the relationship between oxidative stress and uterine fibroids by elucidating the proposed mechanisms and clinical implications.

## 1. Introduction

Despite significant advancements in their diagnosis and treatment, uterine fibroids remain a leading cause of morbidity and altered quality of life among women of reproductive age [1]. The impact of uterine fibroids extends from debilitating symptoms, such as heavy menstrual bleeding and chronic pelvic pain, to the need to perform aggressive surgical interventions to alleviate their effects [1]. Medical treatment, although occasionally effective in the short run, does not provide the permanent relief of symptoms, and many women eventually resort to myomectomy and hysterectomy as definitive treatments [2]. Challenges in tackling uterine fibroids may be related, at least in part, to our incomplete understanding of the pathobiology of these complex tumors, including the mechanisms of their genesis, growth, and symptom development.

**Figure 1:**
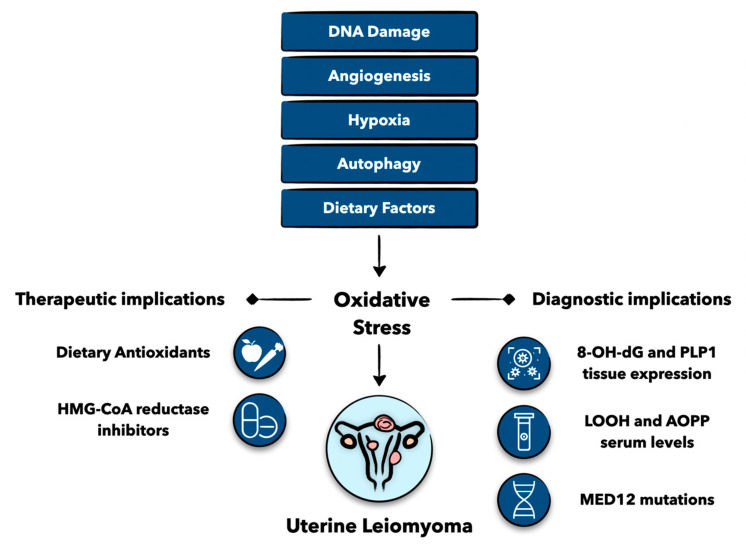
Graphical abstract of various factors leading to oxidative stress and uterine leiomyoma formation. Understanding the relationship between oxidative stress and uterine leiomyomas can lead to important diagnostic and therapeutic implications. Levels of 8-hydroxy-2′-deoxyguonosine (8-OH-dG), proteolipid protein 1 (PLP1), lipid peroxidation products (LOOH), advanced oxidation protein products (AOPP), and mutations in mediator complex 12 (MED12) may be important markers for uterine leiomyoma identification. Dietary antioxidants and 3-hydroxy-3-methyl-gutaryl-coenzyme A (HMG-CoA) reductase have promising therapeutic potential for women with uterine leiomyomas. Figure created with Keynote.

Accumulating research has revealed that there is more to uterine fibroids than their neoplastic properties, and in fact, they possess various and equally important aspects of their genesis, including inflammation, vascular aberrancies, and remodeling [3,4]. Intriguingly, an increasing body of evidence now suggests the contribution of oxidative stress to uterine fibroid development [5,6]. Oxidative stress is the imbalance between prooxidants and antioxidants, and this imbalance is controlled by interconnecting cascades that involve angiogenesis, hypoxia, dietary factors, and smoking/environmental exposures, among others [7,8,9,10]. Oxidative stress in turn drives fibroid development through its impact on genetics, epigenetics, and fibrogenesis [11,12,13]. Further exploring the relationship between oxidative stress and fibroids not only would enhance our understanding of fibroid pathobiology but also could introduce and establish several diagnostic and therapeutic implications specifically designed to target this association (Figure 1).

While several studies have examined different avenues in the role of oxidative stress in uterine fibroids, the majority of evidence has not been presented in a collective piece of literature. In this review, we strive to lay out the multidimensional aspects of the relationship between oxidative stress and uterine fibroids, debate the underlying mechanisms that drive this relationship, and propose several clinical implications tailored to the context of this relationship.

## 2. Role of Reactive Oxygen Species in Female Reproductive Physiology and Disease

Reactive oxygen species (ROS) have been historically established as crucial players in states of health and disease in various organ beds and tissue microenvironments [14]. Essentially, ROS represent a myriad of prooxidant molecules called free radicals, and chief among them are the superoxide anion radical (O^2−^), hydrogen peroxide (H_2_O_2_), and the hydroxyl radical (OH) [15]. Normally, the actions of prooxidant molecules are tightly regulated by antioxidants, achieving an intricate balance that preserves tissue homeostasis, but this balance can be disrupted if ROS exist in overabundance, resulting in oxidative stress [14]. In the female reproductive system, ROS may have important roles in regulating the ovarian cycle and facilitating endometrial shedding during menstruation [16,17], although the precise mechanistic aspects remain to be investigated. The exact cell populations and cellular cascades responsible for ROS production in the female reproductive system remain unknown, but several studies have found that ROS, antioxidants, and their genes are present in various reproductive milieus, including follicular and tubal fluids, oocytes, and embryos [18,19].

At the endometrial level, ROS and superoxide dismutase (SOD), an antioxidant enzyme, may have pertinent roles in triggering endometrial breakdown, with their levels varying during different phases of the endometrial cycle [20]. As demonstrated by Sugino et al., estrogen and progesterone withdrawal at the end of the secretory phase may upregulate ROS and downregulate SOD, with the former triggering a sequential cascade that starts by activating nuclear factor kappa-light chain enhancer of activated B cells (NF-κB) and cyclooxygenase-2 and ends with prostaglandin F_2α_ production, culminating in endometrial shedding [17].

ROS, when existing out of proportion to antioxidants, have been implicated in the development of serious and debilitating reproductive disorders, namely, infertility, endometriosis, and the focus of this review, uterine fibroids [5]. Their injurious properties are manifested through different mechanisms, most notably peroxidative DNA damage, the induction of apoptosis, and lipid peroxidation [21]. The uterus specifically is exposed to various factors related to changes in lifestyle, food, illness, medications, travel, stress, exercise, smoking, heavy metal exposure, radiation, pregnancy, menstruation, and hormonal imbalances [9,22,23]. Many of these factors can lead to oxidative stress and various forms of DNA damage, including single- and double-stranded breaks, abasic sites, base damages, inter-DNA crosslinks, bulky DNA adducts, or mismatched bases, in myometrial cells [24,25], potentially predisposing the uterus to fibroid development. In fact, these tumors are now shown to possess an inherent imbalance between the pro- and antioxidant cellular machinery, creating local milieus of oxidative stress [7]. Having aberrant vasculature and being hormone-responsive, uterine fibroids are further unique in their makeup and growth patterns, establishing a niche where the impact of oxidative stress is modulated by hypoxia and sex steroid hormones [5], as the next sections will elaborate.

## 3. Pathophysiological Considerations

### 3.1. DNA Damage and Genetics

Whole-genome sequencing has demonstrated that a mutation in mediator complex subunit 12 (*MED12*) is present in up to 70% of uterine fibroids and that there is an association between the rate of *MED12* mutations and the tumor number [12,26]. A study performed by Li and colleagues revealed, for the first time, the role of ROS in promoting *MED12* mutations and fibroid development in vitro [12]. As another study showed, human fibroid (Stro−1+/CD44+) stem cells harbor *MED12* mutations due to increased DNA double-stranded breaks and decreased recruitment of DNA repair proteins [24]. Perhaps, one culprit of *MED12* mutations and failed DNA repair could be overexposure to oxidative stress.

From a wider perspective, an increased ROS burden can lead to uncontrolled cellular proliferation, culminating in DNA perturbation and an increased risk of cancer [27]. For example, RAD51 recombinase (*RAD51*) and Breast cancer type 1 (*BRCA1*), genes involved in double-stranded break homologous recombination, are linked to the development of ovarian and breast cancers [27,28] and are also downregulated in uterine fibroids [29]. In ovarian cancer cells, the depletion and inhibition of *RAD51* lead to a significant increase in ROS, particularly in the mitochondria, underscoring the role of *RAD51* in regulating mitochondrial oxidative stress [28]. In breast cancer cells, *BRCA1* functions as a tumor suppressor gene that, when downregulated, leads to cellular proliferation and the accumulation of cellular ROS [27]. Though the relationship between the downregulation of *RAD51* and BRCA1 and increased oxidative stress has not been well studied in uterine fibroids, a similar relationship to the one seen in cancer is likely. Future studies may therefore extrapolate from the cancer literature to help elucidate the potential relationship between oxidative stress and DNA damage in uterine fibroid development.

### 3.2. Hypoxia and Angiogenesis

The abnormal vasculature observed in uterine fibroids and driven by angiogenesis can lead to hypoxic microenvironments, especially in the fibroid core [30]. Generally, hypoxia itself can trigger angiogenesis by upregulating vascular endothelial growth factor (VEGF), hypoxia-inducible factor 1α (HIF1α), T-cell intracellular antigen 1 (TIA1), eukaryotic translation initiation factor 2α (elF2α), and thrombospondin (TSP1) [31]. However, there is conflicting evidence as to whether these hypoxia markers are expressed in uterine fibroids. While Mayer et al. showed the absence of hypoxia-associated markers such as HIF1α/HIF12α, glucose transporter-1 (GLUT-1), and carbonic anhydrase (CA-IX) in in vitro fibroid tissue [32], Miyashita-Ishiwaya et al. demonstrated that hypoxia indeed induces HIF1-α-mediated activation of *VEGF-A*, Adrenomedullin (*ADM*), and Endothelin 1 (*ET1*) genes in fibroid cells [33]. They additionally demonstrated that fibroid cells could proliferate in hypoxic conditions, unlike normal myometrial cells [33].

**Figure 2:**
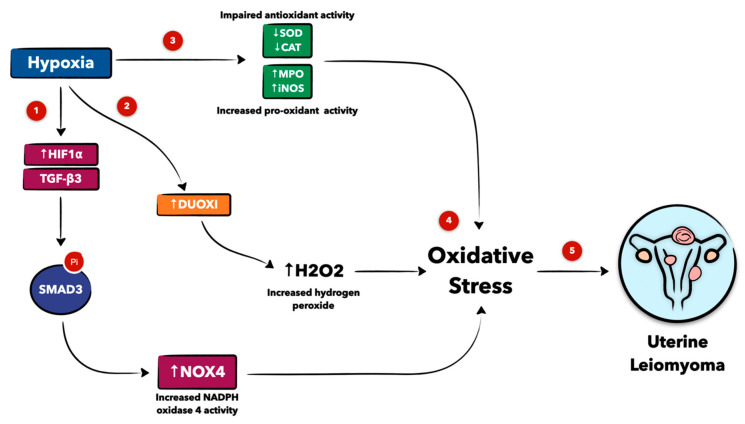
Schematic presentation of the role of hypoxia in uterine leiomyoma development. Hypoxia increases hypoxia-inducible factor 1α (HIF1α) and transforming growth factor-β3 (TGF-β3) expression, leading to the phosphorylation of SMAD family member 3 (Smad3) and the elevated expression of NADPH oxidase 4 (NOX4), ultimately resulting in oxidative stress (1,4). Hypoxia also upregulates dual oxidase (DUOXI) expression, which increases hydrogen peroxide (H_2_O_2_) levels and causes oxidative stress (2,4). Hypoxia also impairs the antioxidant activity of superoxide dismutase (SOD) and catalase (CAT) and promotes the prooxidant levels of myeloperoxidase (MPO) and inducible nitric oxide synthase (iNOS), leading to oxidative stress (3,4). The high degree of oxidative stress produced from hypoxia-induced changes in these pathways contributes to uterine leiomyoma formation (4,5). Figure created with Keynote.

The low-oxygen microenvironment in fibroids is subject to hypoxia-related oxidative stress and impaired antioxidant cellular enzymatic reactions [7]. For example, mRNA activity levels of SOD and catalase (CAT), an antioxidant enzyme that catalyzes the breakdown of hydrogen peroxide to oxygen and water, were compared in human fibroid cell lines and normal myometrial cells with and without hypoxia treatment [7]. The results show a decrease in baseline SOD and CAT expression in human fibroid cells compared to myometrial cells without hypoxic treatment. After hypoxic exposure, SOD and CAT mRNA expression in human fibroid cells was persistently suppressed compared to normal myometrial cells, suggesting an inherent impairment in the antioxidant machinery in uterine fibroids that may be further accentuated under hypoxia (Figure 2) [7]. Hypoxia also has a role in promoting the expression of prooxidant enzymes such as myeloperoxidase (MPO) and inducible nitric oxide synthase (iNOS) in human uterine fibroid cells, leading to decreased apoptosis [7,34]. Fletcher et al. concluded that fibroid cells have increased levels of the oxidative stress markers MPO and iNOS, which are further increased under hypoxic conditions (Figure 2) [5].

Furthermore, hypoxia can have proliferative effects on human fibroid cells by inducing the expression of NADPH oxidase 4 (NOX4) through the HIF-1α/TGF-β3/Smad3/NOX4 signaling pathway, wherein the phosphorylation of Smad3 by TGF-β3 under hypoxic conditions leads to cellular proliferation and fibroid growth (Figure 2) [8]. The NOX family creates superoxide ions, inducing oxidative stress in cells [35]. NOX4 mRNA and protein levels are significantly higher in fibroids compared to myometrial cells when exposed to hypoxic environments [8,35]. Similarly, NOX4 has been shown to be overexpressed in various tumor types and implicated in cellular proliferation and survival [35]. In addition, hypoxia induces the expression of dual oxidase (DUOXI), an enzyme that creates H_2_O_2_ (Figure 2) [35]. Moreover, studies highlighted that the AKT signaling pathway is important to fibroid development under hypoxic environments and seems to be activated by oxidative stress [13].

### 3.3. Autophagy

Autophagy is an important cellular process that involves the sequestration of cytoplasmic organelles and macromolecules in double-membrane autophagosomes in response to nutrient deprivation, viral infections, and genotoxic insults [36]. In various stress-driven conditions, this catabolic process assists in maintaining cellular homeostasis by recycling no-longer-functional cellular components to sustain protein synthesis and metabolism. Research has shown the impact of oxidative stress on autophagy with ROS and reactive nitrogen species (RNS), acting as important intracellular signal transducers to sustain autophagy [36]. Autophagy has specifically been shown to occur in uterine fibroids [11]. There are higher levels of microtubule-associated proteins 1A/1B light chain 3B (LC3), an autophagy marker, and malondialdehyde (MAD), a marker of oxidative stress, in fibroid tissue compared to normal myometrial tissue [11]. Autophagy markers also showed significant positive correlations with MAD and inflammatory cytokines such as TNF-α and TGF-β3 [11]. These results support that oxidative stress, autophagy dysregulation, and inflammation may contribute to the development of uterine fibroids in women [11].

Though some studies indicate the role of increased autophagy in uterine fibroid genesis, other studies indicate that it is the downregulation of autophagy that leads to uterine fibroid development. It is theorized that the downregulation of autophagy is related to the loss of heterozygosity across fumarate hydrase (FH) mutations that are found in uterine fibroids [35]. Fibroids deficient in FH exhibit an impairment in oxidative phosphorylation and a resultant metabolic shift to aerobic glycolysis [37]. In addition, FH mutations attenuate fumarase homotetramers, resulting in the upregulation of the mammalian target of rapamycin (mTOR) and HIF-1α and the downregulation of autophagy activity [38]. This process leads to rapid oncogenic proliferation events such as uterine fibroids [38]. As previously mentioned in this review, oxidative stress can possibly intersect with some of these pathways, potentially contributing to the development of uterine fibroids.

### 3.4. Obesity and Oxidative Stress

Evidence elucidates that obese women are more likely to suffer from several gynecological conditions, such as heavy or irregular menstrual bleeding, endometriosis, infertility, uterine fibroids, and polycystic ovary syndrome [39,40,41,42,43]. The association between obesity and uterine fibroids has been investigated in premenopausal women in several epidemiological studies, with some studies showing an increased risk while others show a nonlinear association [44,45]. Several biological factors have been found to contribute to this association, including altered estrogen metabolism, insulin resistance, and the promotion of inflammation, fibrosis, and angiogenesis [46,47,48,49]. Oxidative stress appears to be a critical component of obesity-related complications and may contribute to the higher risk of uterine fibroids in these women. In a study of premenopausal women with uterine fibroids, Maghraby et al. found increased expression of a fibrosis marker, fibroblast activating protein (FAP), and markers of oxidative stress, inflammation, and autophagy in obese patients compared to non-obese individuals, as stated prior in this review [11]. Moreover, FAP and autophagy markers are significantly correlated with body mass index (BMI) [11]. Chronic inflammation triggered and sustained by visceral fat may play a crucial role in promoting cellular proliferation in uterine fibroids [50]. The increase in visceral fat may explain the changes in cholesterol fractions, which positively correlate with BMI, lipid peroxidation, and oxidized low-density lipoprotein (LDL) [50]. In addition, a diet rich in antioxidants during the reproductive years may reduce the risk of fibroids by mediating the effects of oxidative damage normally seen in overweight and obese individuals. Our group has previously discussed the role of the gut microbiota in mediating obesity-induced inflammation and its systemic complications, but whether this involves oxidative stress or is linked to uterine fibroid development remains to be investigated [39].

### 3.5. Antioxidant Defense

**Figure 3:**
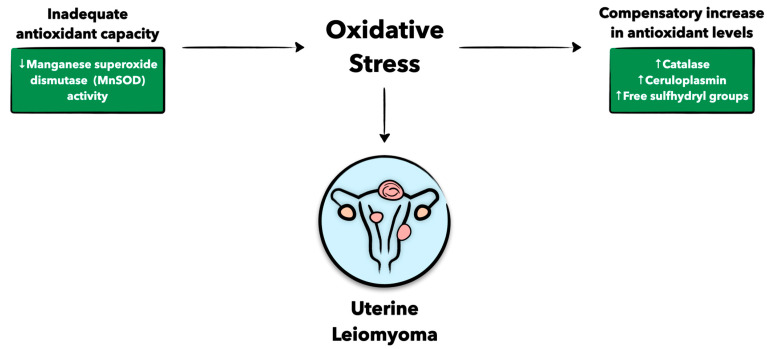
Schematic presentation of the role of antioxidants in uterine fibroid development. Figure created with Keynote.

Antioxidants are essential in counteracting oxidative damage, and without functional antioxidants, oxidative stress can build up, leading to excessive tissue injury (Figure 3). In the context of this review, antioxidant levels seem to be associated with fibroid growth. As one study shows, ceruloplasmin (Cp), catalase (Cat), and free sulfhydryl groups (-SH), all markers of antioxidant capacity, seem to positively correlate with fibroid volume [51]. Additionally, there is an increase in the antioxidant activity of glutathione peroxidase (GPX) in fibroid tissue compared to the normal myometrium [52]. This increase in antioxidants in fibroid tissues is likely a compensatory response to a heightened oxidative burden in these tissues [52]. On the other hand, it has also been shown that uterine fibroids have higher levels of oxidative stress without adequate antioxidant capacity [53,54]. For example, the acetylation of manganese superoxide dismutase (MnSOD) decreases its activity and is observed in 70% of women with fibroids [55], possibly conferring a defective antioxidant response in uterine fibroids. In turn, inactive MnSOD drives oxidative stress while promoting prooxidative proliferation and initiating a downstream effect on AKT activation, potentiating the survival of fibroid cells in a prooxidative environment [55]. While data on the role of antioxidants in uterine fibroid development may be conflicting, defective containment of oxidative stress in these tumors remains highly plausible, and further evidence is warranted to accurately delineate the nature between antioxidants and promoters of oxidative stress in these tumors.

## 4. Clinical Implications

Currently, women can undergo non-surgical and surgical treatments to treat their uterine fibroids. Non-surgical treatments include hormonal therapies such as gonadotropin-releasing hormone analogs (GnRHa), progestins, selective estrogen receptor modulators, dopamine agonists, prostaglandin analogs, and selective progesterone receptor modulators [56,57]. Surgically, women with uterine fibroids can undergo a hysterectomy or non-invasive surgical treatments, including magnetic-resonance-guided focused high-intensity ultrasound, and minimally invasive techniques, including thermal tissue ablation and uterine artery embolization [58,59,60]. Despite the availability of these options, hysterectomies are the most common treatment for uterine fibroids [61]. However, a significant proportion of women with uterine fibroids prefer not to undergo hysterectomies, indicating a clear need for additional interventions for these patients. Pharmaceutical interventions could play a key role in improving treatment options for women with uterine fibroids. Besides hormonal therapies, many antioxidants seem to be effective in both the prevention and treatment of uterine fibroids, as shown in pre-clinical and clinical studies, but additional evidence is still needed [3,4].

### 4.1. Biomarkers as Diagnostic Adjuncts

Determining biomarkers of oxidative stress in women with uterine fibroids can help introduce novel diagnostic and therapeutic options (Table 1). 8-Hydroxy-2′-deoxyguonosine (8-OH-dG) is a commonly used marker of oxidative stress. Though it has yet to be used as a diagnostic tool for uterine fibroids, it has promising potential. Human fibroid tissue shows increased 8-OH-dG levels compared to normal myometrial tissue, with levels correlating with tumor size [62]. Similar results were found in urine samples from women with fibroids [53]. From day 14 to 21 of the menstrual cycle, urine 8-OH-dG levels were higher in women with fibroids than in women without fibroids, possibly suggesting an increased oxidative burden during the luteal phase of menstruation [53]. Immunohistochemical staining supports the association between 8-OH-dG and fibroids, as significantly higher levels are seen in human fibroid biopsy tissue compared to normal myometrial tissue [63]. Moreover, a more recent study examining the gene expression profiles of human fibroid tissues compared to normal myometrial tissue from the Gene Expression Omnibus database determined that proteolipid protein 1 (PLP1), a member of the TNF superfamily, had upregulated mRNA and protein levels in fibroid tissues compared to normal tissues [64]. Therefore, PLP1 could serve as a useful diagnostic tool for identifying patients with uterine fibroids [64].

In addition, lipid peroxidation (LOOH) products, which act as markers of oxidative stress, are elevated in the sera of women with hyperplastic and neoplastic endometrial cells [65]. There is also an increased concentration of carbonyl groups and advanced oxidation protein products (AOPPs) and decreased thiol levels in the sera of women with fibroids compared to those of women without fibroids [6]. The heightened serum AOPP and carbonyl levels may be indicative of the degree of oxidative stress, as oxidation affects the structure of proteins and increases their half-life. In fact, other diseases associated with oxidative stress, such as diabetes mellitus, neurodegenerative disorders, and rheumatoid arthritis, also have elevated serum protein oxidation products [6,66,67,68]. Moreover, thiols exert antioxidant activity and have in fact been shown to be decreased in the sera of women with fibroids, potentially contributing to the development of these tumors [6].

Santulli and colleagues have described the relationship between serum marker levels and the anatomical features and clinical findings related to fibroids [6]. Serum AOPP levels correlate with total fibroid weight and the duration of infertility, whereas serum protein carbonyls correlate with the duration of infertility [6]. These serum markers of oxidative stress can therefore be used to depict information about fibroids that could only previously be garnered through more invasive measures such as biopsies, demonstrating the revolutionary effect serum biomarkers will have on the diagnosis and monitoring of uterine fibroids.

Somatic mutations in MED12, which are implicated in modulating oxidative stress in uterine fibroids, are believed to be reliable biomarkers for these tumors [6,7]. Mutations in MED12 exon 2 were found in 70% of fibroids [8], and MED12m-related fibroids were smaller in size but more numerous compared to the wild type [9]. Additionally, Maekawa et al. revealed that MED12m-related fibroids possessed a rich extracellular matrix but poor vasculature, while wild-type fibroids were characterized by rich vasculature and smooth muscle cell proliferation [8]. Therefore, the altered fibroid phenotype associated with MED12 could help tailor individualized therapies targeted at the unique features of these phenotypes [8].

### 4.2. Dietary Antioxidants

Diet and nutrients were reported to play a role in uterine fibroid development and growth. Food additives such as butylated hydroxytoluene (BHT), an antioxidant additive found in cereals, butter, potato chips, and flavoring agents, may play a role in uterine fibroid development [69]. BHT is added to foods to increase their shelf-life and the stability of fat-soluble vitamins [69]. In mouse models, BHT promoted uterine fibroid formation by affecting P3K/Akt and MAPK signaling pathways, leading to increased proliferation and extracellular matrix accumulation [69]. Though further studies in humans need to be conducted, it is likely that a similar finding will be found. Additionally, exposure to environmental endocrine-disrupting chemicals (EDCs) can lead to uterine fibroid formation [70]. EDCs are chemicals that can interfere with hormones and are found in milk and cheese products, along with other household products [71]. EDCs such as diethylstilbestrol can act as estrogen agonists, which can lead to fibroid development due to its hormonal dependence [70].

A diet low in antioxidants can increase the risk of uterine fibroids, while dietary antioxidants can exert protective effects [72]. Fruit and vegetables are rich in antioxidants and phytochemicals and confer a lower risk of uterine fibroids [7,73,74]. Dietary antioxidants include vitamins, such as vitamins D, A, C, and E, and active compounds, such as polyphenols, curcumin, and indoles, as well as minerals such as selenium [72]. Vitamin D has been studied extensively for its role in the biology and therapy of uterine fibroids and has been suggested as a potential therapy for the prevention and treatment of fibroids [75]. A significant reduction in fibroid size (>30%) was reported in two pilot clinical studies, wherein premenopausal Egyptian and Italian women were treated with green tea extract and/or vitamin D [76,77].

Likewise, vitamin C has major antioxidant properties. Heinonen et al. performed global metabolic profiling of both fibroid and corresponding myometrial samples. In the MED12m fibroid subtype, significant dysregulation of the antioxidant pathway of vitamin C was found in fibroid samples based on both metabolic and gene expression levels [78]. However, more experimental studies are needed to investigate the prophylactic and therapeutic use of vitamin C in uterine fibroids [75]. A vitamin A-enriched diet and synthetic retinoid analogs may also prevent or decrease fibroid growth [72]. It is reported that vitamin A-enriched citrus fruit may decrease fibroid risk through sex-steroid-hormone- or antioxidant-mediated pathways [74]. However, other studies have shown a positive association between vitamin A and fibroid development through vitamin A-mediated activation of fibroid-promoting gene expression via activation of peroxisome proliferator-activated receptors [72], emphasizing the need to further investigate its role in these tumors.

Other dietary factors with potential beneficial effects on fibroids include lycopene, which is a potent antioxidant with a 40-carbon carotenoid found in tomatoes and is thought to reduce the development and growth of uterine fibroids [74]. In addition, vitamin B3 may act as an antifibrotic agent via antioxidant mechanisms and a reduction in TGF-β expression [79]. Similarly, curcumin and dairy products may ameliorate the oxidative burden in uterine fibroids [72,74].

Further, fruit and vegetables rich in carotenoids, polyphenols, quercetin, and indole-3-carbinol have shown possible benefits in uterine fibroid patients [72]. The implicated mechanism may involve proapoptotic, hormone-dependent, anti-inflammatory, anti-proliferative, antifibrotic, and anti-vascular pathways, but further research is needed [73,74].

### 4.3. Statins

Statins are 3-hydroxy-3-methylglutaryl coenzyme A (HMG-CoA) reductase inhibitors mainly used for the treatment of hypercholesterolemia. Statins have shown the ability to inhibit tumor growth in many cancer types, including breast, ovarian, prostate, colon, leukemia, and lung cancers [80]. As our group has previously demonstrated, statins can inhibit the proliferation of human leiomyoma cells, possibly by modulating oxidative stress [81]. Our previous studies have shown a reduction in fibroid risk and symptom development with statin use [82]. Simvastatin, a semisynthetic lipophilic HMG-CoA reductase inhibitor, has demonstrated anti-fibroid properties by inhibiting fibroid cell proliferation, decreasing the expression of proliferative markers such as Ki67, and promoting apoptosis of fibroid cells (in a dose-dependent manner) in in vitro studies [81,83]. Furthermore, simvastatin can reduce the expression of β1 integrin (44%) and type 1 collagen (60%) compared to untreated leiomyoma cells [84]. The possible mechanisms include the amelioration of the stiffness and disordered mechanical homeostasis of fibroids [84] and the modulation of estrogen/estrogen receptor signaling via regulating receptor palmitoylation, trafficking, and degradation in cellular and animal experiments [85]. Currently, an ongoing clinical trial is evaluating the effects of simvastatin in patients with uterine fibroids (IRB Number: IRB00149869).

## 5. Conclusions and Future Directions

For decades, uterine fibroids have been heavily studied, and researchers have uncovered unique perspectives on uterine fibroid pathophysiology. This paper enriches our understanding of how oxidative stress represents a novel and integral feature of uterine fibroid pathobiology, contributing to their development. As we become increasingly aware of this association, curiosity to learn its mechanistic aspects will lead us to previously unexplored pathobiological avenues and introduce novel frameworks that will have future diagnostic and therapeutic implications. Therefore, while this paper comprehensively explores this association, it also emphasizes the need for further experimental and clinical research to delve into the cellular and molecular mechanisms related to oxidative stress in these chronic, costly, and debilitating tumors.

## Figures and Tables

**Table 1:** Table of biomarkers and their uses in women with uterine fibroids.

Biomarker	Diagnostic Usage
8-OH-dG	Protein found in fibroid tissue; levels correlated with tumor size; can be found in urine
PLP1	mRNA and protein found in fibroid tissues
LOOH	Elevated in the sera of women with hyperplastic and neoplastic endometrial cells
Carbonyl groups and AOPPs	Increased in sera of women with fibroids
Thiols	Decreased in sera of women with fibroids
MED12	Somatic mutation present in 70% of fibroids

## Data Availability

The data is contained within the article.

## Abbreviations

8-OH-dG	8-hydroxy-2′-deoxyguonosine
ADM	Adrenomedullin
AOPP	Advanced oxidation protein products
BHT	Butylated hydroxytoluene
BMI	Body mass index
BRCA1	Breast cancer type 1
CA-IX	Carbonic anhydrase
CAT	Catalase
Cp	Ceruloplasmin
DUOXI	Dual oxidase
EDC	Endocrine-disrupting chemicals
elF2a	Eukaryotic translation initiation factor 2a
ET1	Endothelin 1
FAP	Fibroblast activation protein
FH	Fumarate hydrase
GLUT-1	Glucose transporter-1
GnRHa	Gonadotropin-releasing hormone analogues
GPX	Glutathione peroxidase
H_2_O_2_	Hydrogen peroxide
HIF1a	Hypoxia inducible factor 1α
HMG-CoA	3-hydroxy-3-methylglutaryl coenzyme A
iNOS	Nitric oxide synthase
LC3	Microtubule-associated proteins 1A/1B light chain 3B
LDL	Low-density lipoprotein
LOOH	Lipid peroxidation products
MAD	Malondialdehyde
MED12	Mediatory complex subunit 12
MnSOD	Manganese superoxide dismutase
MPO	Myeloperoxidase
mTOR	Mammalian target of rapamycin
NOX4	NADPH oxidase 4
O_2_^−•^	Superoxide anion radical
OH^•^	Hydroxyl radical
PLP1	Proteolipid protein 1
RAD51	RAD51 recombinase
RNS	Reactive nitrogen species
ROS	Reactive oxygen Species
SH	Free sulfhydryl groups
Smad3	SMAD family member 3
SOD	Superoxide dismutase
TGF-b3	Transforming growth factor-β3
TIA1	T-cell intracellular antigen 1
TNFα	Tumor necrosis factor α
TSP1	Thrombospondin
VEGF	Vascular endothelial growth factor
